# West Nile Virus Epidemic, Northeast Ohio, 2002

**DOI:** 10.3201/eid1111.040933

**Published:** 2005-11

**Authors:** Anna M. Mandalakas, Christopher Kippes, Joseph Sedransk, Jeffery R. Kile, Asha Garg, John McLeod, Richard L. Berry, Anthony A. Marfin

**Affiliations:** * Case Western Reserve University, Cleveland, Ohio, USA; † Cuyahoga County Board of Health, Cleveland, Ohio, USA; ‡ Ohio Department of Health, Columbus, Ohio, USA; § Centers for Disease Control and Prevention, Fort Collins, Colorado, USA

**Keywords:** West Nile Virus, Seroepidemiologic Study, Seroprevalence, Risk Factors, Arboviruses, Flaviviridae, dispatch

## Abstract

Serum samples and sociodemographic data were obtained from 1,209 Ohio residents. West Nile virus immunoglobulin M (IgM) and IgG antibodies were detected by enzyme-linked immunosorbent assay and confirmed. Children were 4.5 times more likely to become infected yet 110× less likely to have neuroinvasive disease develop.

Since its 1999 North American introduction, West Nile virus (WNV) has emerged as an important cause of illness and death. Although several at-risk populations have been identified, older age remains the major risk factor for developing encephalitis after infection (*1**–**4*).

WNV rapidly spread across the United States, resulting in intense epidemic activity in Louisiana, Illinois, Michigan, and Ohio in 2002; Colorado in 2003; and Arizona and California in 2004 (*5**,**6*). In Ohio, WNV infections were first recognized in animals in 2001. In 2002, Ohio reported 341 human cases of WNV encephalitis or meningitis (West Nile neuroinvasive disease [WNND], incidence: 28 cases/million population) with 31 deaths. In 2002, Cleveland and surrounding Cuyahoga County (2000 population 1,393,978 of whom 1,302,982 were >5 years of age) reported 221 laboratory-confirmed cases of WNV illness, including 155 WNND cases (111 cases/million population) with 11 deaths from July 30 to October 3. All reported WNND patients (median age 61 years, range 11–98 years) were hospitalized (CDC ArboNET Surveillance Network, unpub. data).

Since most WNV infections are asymptomatic (*7**,**8*), the true rate of WNV infection can best be estimated by measuring the prevalence of WNV-specific antibody in a recently exposed population. In December 2002, the Cuyahoga County public health community conducted a household-based seroprevalence survey to estimate neighborhood and countywide WNV infection rates.

## The Study

The survey was conducted December 5–12, 2002. Stratified multistage cluster sampling was used to estimate countywide and subpopulation prevalence rates. The county was divided into 3 risk strata (Table 1). Census tracts were sampled within strata with probability proportional to population. Within each census tract, clusters of ≈50 households were formed. At random points, residents were approached for recruitment until 10 participating households were enrolled from each cluster.

**Table 1:** WNV seroprevalence*

Seroprevalence	No. positive/no. tested	Weighted % (95% CI)
Overall	34/1,209	1.9 (0.8–4.6)
Age-specific		
5–17 y	4/168	6.5 (4.3–9.5)
18–64 y	25/790	1.3 (0.4–4.5)†
>65 y	5/219	1.4 (0.4–4.5)‡
Strata-specific		
More human illnesses reported; higher MIR(stratum 1)§¶	16/463	2.5 (0.6–9.2)
Fewer human illnesses reported; varying MIR (stratum 2)#	7/453	1.5 (0.2–4.4)
No human illnesses reported; varying MIR (stratum 3)**	11/293	3.3 (0.4–23.9)

*WNV, West Nile virus; CI, confidence interval; MIR, minimum infection rate. †Significant difference between 5- to 17-year-old and 18- to 64-year-old patients (p<0.02). ‡Significant difference between 5- to 17-year-old and >65-year-old patients (p<0.01). §Reference 9. ¶Stratum 1 included neighborhoods with at least 9 reported human cases, a WNV case rate >4.5/10,000, and mosquito MIR >15/1,000. #Stratum 2 included neighborhoods with at least 1 reported human case, a WNV case rate <4.5/10,000, and varying levels of MIR (0–54/1,000). **Stratum 3 included neighborhoods with no known human cases and varying levels of MIR.

Residents >5 years of age who had lived in the household since July 1, 2002, were asked to participate by providing a blood sample and responding to a questionnaire. One person from each household completed a questionnaire about the home environment. Questionnaires developed by the Centers for Disease Control and Prevention (CDC) were used (*10*). Informed consent was obtained from all participants or their legal guardian. Assent was obtained from minors >8 years of age. Residents were offered a US $10 gift certificate and test results as compensation. Persons who were pregnant, mentally handicapped, or taking anticoagulants were not enrolled. Institutional review board approval was obtained from University Hospitals of Cleveland.

Serum samples were screened with a WNV-specific immunoglobulin M (IgM) antibody-capture (MAC) enzyme-linked immunosorbent assay (ELISA) (11) and indirect IgG ELISA at Focus Laboratories (Cypress, CA, USA). Positive IgM and IgG were defined as an antibody index >2.0 and >0.9, respectively. All IgM- and IgG-positive samples were sent to the Viral and Rickettsial Laboratory, California Department of Health Services (Richmond, CA, USA) for confirmatory plaque reduction neutralization tests to identify WNV and St. Louis encephalitis virus (SLEV)–specific neutralizing antibody. At the second laboratory, WNV MAC-ELISAs (*12*) were repeated and IgG ELISAs for WNV, SLEV, and dengue were performed (*13*). Laboratory-based case definitions were developed (Table 2).

**Table 2:** Laboratory-based definitions used for confirmatory testing*†‡

Case	Definition
Confirmed WNV infection	WNV IgM MAC-ELISA positive and WNV PRNT titer >1:20 and WNV PRNT titer >2-fold than SLEV PRNT titer **or**
	WNV PRNT titer >1:20 and WNV PRNT titer >4-fold than SLEV PRNT titer
Probable WNV infection	WNV PRNT titer >2-fold than SLEV PRNT titer
Previous SLEV infection	SLEV PRNT titer >1:20 and SLEV PRNT titer >2-fold than WNV PRNT titer
Probable nonspecific flavivirus infection	Negative WNV and SLEV PRNT results **and**
	Negative WNV IgM MAC-ELISA results **and**
	Positive WNV, SLEV, or dengue IgG EIA results **and**
	No history of YFV or JEV vaccination
Previous infection	History of YFV or JEV vaccination, WNV IgM MAC-ELISA negative, and WNV and SLEV PRNT negative

*WNV, West Nile virus; IgM, immunoglobulin M; MAC-ELISA, IgM antibody capture enzyme-linked immunosorbent assay; PRNT, plaque reduction neutralization test; SLEV, St. Louis encephalitis virus; EIA, enzyme immunoassay; YFV, Yellow fever virus; and JEV, Japanese encephalitis virus. †All specimens referred for confirmatory testing were positive for WNV IgG during initial screening. ‡Case definitions were developed in consultation with the Centers for Disease Controla nd Prevention and the Ohio Department of Health.

SPSS version 11.5 (SPSS Inc, Chicago, IL, USA) and SUDAAN version 8.0 (Research Triangle Institute, Research Triangle Park, NC, USA) were used for preliminary analyses and to assess differences in demographics, behavior, and clinical characteristics between seropositive and seronegative persons. Since SUDAAN variance estimation did not accommodate our complex sample design, we developed formulas to provide better estimates of variance and confidence intervals (CIs) using an α = 0.05. Unless noted, all analyses were weighted. Individual weights were derived by taking the inverse of the probability of selection.

The standard Horvitz-Thompson estimator was used for point estimation (*14*). For variance estimates, all sources of variation that resulted from the selection process were included by using standard Taylor series approximations. To calculate the confidence interval for the true prevalence ratio (PR), we approximated the variance of the logarithm of the sample PR by using standard Taylor series method. The end points of this interval were exponentiated to obtain the interval for PR.

## Conclusions

Participants were recruited from 13 Cuyahoga County municipalities and 9 Cleveland neighborhoods. Of 4,676 households visited, 2,318 households had an eligible adult present; of these eligible households, 819 households (35.3%) agreed to participate. Of 1,747 eligible residents in 819 households, 1,251 (71.6%) consented to participate; 42 participants in 13 households had insufficient serum samples and were excluded. The study sample consisted of 1,209 participants from 806 households; they had a mean age of 43.2 years (range 5–94 years) and included 168 (12.4%) children 5–17 years of age. Compared to 2000 Cuyahoga County census demographics, our study sample contained a significantly larger proportion of adults 18–64 years of age (75.7% vs. 63.5%), female participants (57.8% vs. 52.8%), and African Americans (31.8% vs. 27.4%).

Initial screening identified WNV IgM and IgG antibody in 4 serum samples, IgG only in 90 serum samples, and IgM only in 2 specimens. Based on criteria listed in the Table 2, confirmatory testing of the 96 samples identified 27 confirmed and 7 probable WNV-infected persons. The countywide seroprevalence rate was 1.9% (95% CI 0.8–4.6) (Table 1), which suggests that 10,400–59,900 residents were infected. Based on 155 WNND cases reported from Cuyahoga County, ≈1 WNND case occurred for every 160 infected persons (95% CI 1:67–1:386).

Seroprevalence varied significantly between age groups (p<0.05) (Table 1). Based on reported WNND cases and age-stratified seroprevalence rates, we estimate that 1 case of WNND occurred per 4,167 infected children 5–17 years of age, per 154 infected adults 18–64 years of age, and per 38 infected persons >65 years of age (Figure). Strata-specific seroprevalence values ranged from 1.5% to 3.3% but were not statistically different (Table 1).

**Figure:**
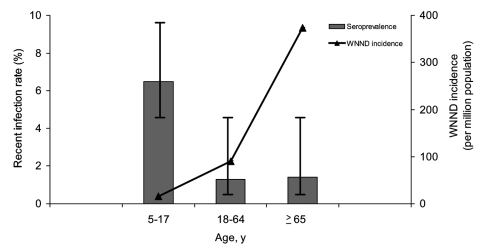
Comparison of age-stratified seroprevalence rates (gray bars) to the age-stratified incidence of West Nile neuroinvasive disease (WNND) (black line). Seroprevalence rates were measured in the 2002 seroprevalence study. The incidence of WNND was based on cases reported through the local disease reporting system during the 2002 transmission season.

In 2002, Cuyahoga County experienced its largest epidemic of arboviral encephalitis and meningitis, yet only 1.9% of the county's population became infected during this first WNV transmission season. In the 733-km^2^ area of Cuyahoga County, 155 cases of encephalitis and meningitis (WNND incidence: 111 cases/million population) occurred; the seroprevalence was 1.9% countywide and 2.5% in the selected highest risk survey stratum.

Little is known about WNV infection rates in children (*15*). In contrast to a previous study (*8*), our study demonstrated an age-dependent risk for WNV infection. The antibody prevalence in the 5- to 17-year age group was significantly greater than in older age groups. These data suggest that children were 4.5 times more likely to be infected than older persons. In this study, children reported spending more time outdoors and using less personal protective measures, which likely contributed to their higher seroprevalence rate. In 2002, only 4 cases of WNND were reported in the 5- to 17-year age group, resulting in a WNND:infection ratio of 1:4,200 compared to a 1:38 ratio among persons >65 years of age. Thus, the risk for WNND after infection may be as much as 110× greater in adults >65 years of age, as compared to children. Inclusion of a larger number of children in this study compared to previous studies allowed these age-stratified analyses to be completed.

Although WNV seroprevalence was similar to those measured in previous outbreaks (7,8), our study was the first to demonstrate that the risk for WNV infection can be age-dependent. Children had a higher rate of infection than adults, but serious neurologic disease developed in few of them. This finding has implications for public health practice and emphasizes the need for children to use protective measures to prevent mosquito bites to further lower their risk for infection with WNV and other mosquitoborne viruses.
